# Phthalate exposure induces cell death and ferroptosis in neonatal microglial cells

**DOI:** 10.55730/1300-0144.5889

**Published:** 2024-06-02

**Authors:** Elif KELEŞ, Arzu ARAL, Zübeyir ELMAZOĞLU, Hasan Hüseyin KAZAN, Elif Gülçiçek ABBASOĞLU TOPA, Mehmet Ali ERGÜN, Hayrunnisa BOLAY

**Affiliations:** 1 Neuropsychiatry of Education, Research and Application Center, Faculty of Medicine, Gazi University, Ankara, Turkiye; 2 Department of Radiology, Feinberg School of Medicine, Northwestern University, Chicago, USA; 3 Neuroscience and Neurotechnology Center of Excellence (NÖROM), Ankara, Turkiye; 4 Department of Immunology, Faculty of Medicine, Yeditepe University, İstanbul, Turkiye; 5 Department of Pharmacology, Faculty of Pharmacy, Ankara Medipol University, Ankara, Turkiye; 6 Department of Medical Biology, Gülhane Faculty of Medicine, University of Health Sciences, Ankara, Turkiye; 7 Department of Medical Genetics, Faculty of Medicine, Gazi University, Ankara, Turkiye; 8 Department of Neurology, Faculty of Medicine, Gazi University, Ankara, Turkiye

**Keywords:** Phthalate, DEHP, microglia, inflammation, ferroptosis

## Abstract

**Background/aim:**

Phthalates are the materials used for plasticizing polyvinyl chloride. Di-(2-Ethylhexyl) phthalate (DEHP) is one of the phthalates most frequently used in a wide range of applications, including medical equipment such as endotracheal and feeding tubes, intravenous catheters, central lines, extracorporeal membrane oxygenation sets, total parenteral nutrition bags, blood product sets, and intravenous pump lines, respiratory sets in neonatal intensive care units (NICUs). Studies have shown that phthalates, including DEHP, can cross the placenta and blood-brain barrier, possibly leading to neurodevelopmental impairment in vitro and in vivo. However, the molecular mechanisms affected by phthalate exposure have not been explored in depth. This study aimed to illuminate the effects of DEHP on neuroinflammation at the molecular level using neonatal microglial cells as the model.

**Materials and methods:**

Mouse BV-2 neonatal microglia cells were exposed to DEHP under controlled conditions. Cellular toxicity was assessed via a cell viability assay and specific markers were used to evaluate the apoptosis/necrosis, cellular iron content, reactive oxygen species (ROS), and organelle integrity. Proinflammatory proteins were quantified using enzyme-linked immunosorbent assay, while ferroptosis was assessed using a ferroptosis blocker, and affected gene expressions were determined using quantitative reverse-transcriptase real-time polymerase chain reaction (RT-PCR).

**Results:**

The results revealed that high concentrations of DEHP exposure increased toxicity via increased levels of ROS and inflammation. Elevated ROS levels were observed to increase the tendency for mitochondrial-lysosomal disruption, bringing about apoptosis or necrosis. Moreover, iron homeostasis was dysregulated by DEHP, which putatively triggered ferroptosis in a dose-dependent manner.

**Conclusion:**

This study indicates that neonatal exposure to DEHP may be linked to neurodevelopmental impairment via inflammation-related cell death and ferroptosis. The prevalence of DEHP in NICU medical devices raises concerns about potential neurodevelopmental deficits, including disorders like autism and mental retardation. These findings highlight the urgency of addressing DEHP exposure in neonatal care.

## 1. Introduction

Phthalates are synthetic compounds and esters of phthalic acids. They are widely used in industry as they advance the plasticity of industrial polymers [1]. Among them, di(2-ethylhexyl) phthalate (DEHP) is prominently used for the plasticization of polyvinyl chloride (PVC). Thus, it is found in a wide range of daily materials, including clothing, toys, and packaging, in addition to medical equipment [2].

Owing to frequent preferences for plastic materials, humans may be extensively exposed to DEHP. This exposure could be by dermal, oral, or intravenous routes or by inhalation. In humans, DEHP has been shown to be extremely toxic, damaging developmental stages, disrupting multiple organs and systems, and causing genotoxicity and/or carcinogenesis due to its metabolism into oxidative compounds [3]. Phthalates can pass through the placenta and blood-brain barrier [4] and could be transferred to newborns by breast milk [5]. During the neonatal period, infants who are admitted to the neonatal intensive care unit (NICU) are exposed to various medical devices, including intravenous catheters, respiratory sets, masks, total parenteral nutrition bags, transfusion sets, feeding tubes that contain plasticizers, which could have an impact on their developmentally vulnerable state [6]. Additionally, DEHP exposure via medical equipment could be high in infants in the NICU [3,6].

Microglia are immune cells that play a crucial role in the inflammatory response of the central nervous system (CNS). They comprise approximately 10%–15% of the total population of brain cells. Despite the highly regulated nature of these cells, any deviations in their functions can trigger neurodegeneration and brain injuries during the perinatal and neonatal stages [7]. Importantly, microglia regulate cell death mechanisms via metabolic signals [8], which make reactive oxygen species (ROS) critical. ROS have been determined as the reason for the microglial function impairment caused by DEHP exposure [9]. ROS-mediated microglial activation and neuroinflammation, as a result, have generally been linked to specific cell death mechanisms, including apoptosis, pyroptosis, and ferroptosis, through diverse molecular mechanisms [10,11]. Despite extensive research on the impact of phthalates on microglial activation, there remains a lack of understanding regarding the cumulative molecular mechanisms that are changed during exposure.

The present study aimed to identify in detail the molecular mechanisms altered in the neonatal microglia following phthalate exposure, particularly DEHP exposure. BV-2 neonatal microglial cells were used to investigate the potential mechanisms involved in inflammatory processes on the axis of ferroptosis-apoptosis/necrosis-oxidative stress after DEHP exposure.

## 2. Materials and methods

### 2.1. Cell culture

Mouse BV-2 neonatal microglia were cultured in DMEM/F-12 medium (Sigma-Aldrich Chemical Co., St. Louis, MO, USA) in the presence of 10% fetal bovine serum (FBS), 1% glutamine, penicillin/streptomycin, and amphotericin B (Capricorn Scientific GmbH, Germany) at 37 °C and 5% CO_2_. Cells were passaged or seeded when the confluency was 80%.

### 2.2. DEHP treatment and cell viability assessment

3-(4,5-Dimethylthiazol-2-yl)-2,5-diphenyltetrazolium bromide (MTT; Biological Industries, Beit HaEmek, Israel) was used for the viability assay. Briefly, 2 × 10^4^/well cells were seeded into the wells of a 96-well plate and incubated overnight. After washing with PBS, the cells were treated with 1 nM to 10 mM of DEHP, which was partially dissolved in dimethyl sulfoxide (DMSO) and diluted with cell culture medium. The concentrations were clarified according to the literature for determined durations in the presence of solvent-only control groups [3,12–15]. Next, 0.5 mg/mL of MTT solution dissolved in PBS was added into the medium and incubated at 37 °C for 4 h. Finally, the cells were disrupted with 100 μL of 10% sodium dodecyl sulfate containing 0.01 N of hydrochloric acid (HCl) and incubated overnight. Optical densities (ODs) were determined by Sunrise Plate Reader (Tecan, Austria) at 490 and 570 nm. The OD value of the control group was fixed at 100%, and the treated ones were correlated [16].

### 2.3. Apoptosis/necrosis ratio

Apoptosis and necrosis were assessed through acridine orange (AO) and ethidium bromide (EtBr) staining [17]. As described above, the cells were seeded and incubated with DEHP or the solvent. After washing, the cells were incubated with a mixture of AO (1 μg/mL; Thermo Fisher Scientific Inc., Waltham, MA, USA) and EtBr (1 μg/mL; Thermo Fisher Scientific Inc.) for 30 min at 37 °C. Images were captured using an LSM 900 confocal microscope (Carl Zeiss AG, Oberkochen, Baden-Württemberg, Germany) under the wavelengths of 500/525 (excitation (ex)/emission (em); AO) and 530/617 nm (ex/em; EtBr) from at least four different regions of the wells. Fluorescence quantification was performed using ImageJ (US National Institutes of Health, Bethesda, MD, USA), where the fluorescence in the selected area was evaluated as integrated fluorescence integrity, normalized to the nuclei staining dye, and presented as integrated relative fluorescence units (RFUs) [18].

### 2.4. Cellular iron contents

Intracellular total and ferrous (Fe^2+^) iron levels were determined via Prussian blue staining (Sigma-Aldrich Chemical Co.) [19]. Cells were seeded into the wells of a 96-well plate and treated with DEHP solution for specific durations, as described above. After incubation, the cells were treated with 1) 10 mM ascorbic acid in 1X PBS and 4% potassium ferrocyanide: 12% HCl (1:1) for the total iron levels, or 2) only 4% potassium ferrocyanide: 12% HCl (1:1) for the free iron for 1 h at room temperature. Finally, ODs were obtained at 593 and 700 nm separately for the total and free iron measurements, and the results were compared by comparing them to that of the control group 100%.

In addition to the Prussian blue staining and detection of the total and free iron levels, Calcein-AM (Thermo Fisher Scientific Inc.) was used to stain the intracellular labile iron pool (LIP). As described above, the cells were seeded and incubated with DEHP in the solvent-only control group. Next, the cells were fixed using 4% paraformaldehyde and incubated with 5 μM of Calcein-AM for 30 min at 37 °C [20]. Then, after washing with 1X PBS, the cells were further treated with Hoechst 33342 (1 μg/mL) for 5 min at room temperature. After washing, images were obtained at 361/497 nm (ex/em; Hoechst) and 488/530 nm (ex/em; Calcein-AM), as explained above.

### 2.5. Intracellular ROS levels

Intracellular ROS levels were determined using 2′,7′-diclorofluoresin diacetate (DCFH-DA) [21]. Briefly, the cells were seeded and incubated with DEHP in the presence of a solvent-only control group, as described above. Next, the cells were washed and incubated with DCFH-DA (20 μM; Sigma-Aldrich Chemical Co.) and Hoechst 33342 (1 μg/mL; Thermo Fisher Scientific Inc.) probes for 30 min. After washing, images were obtained at 361/497 nm (ex/em) and 488/535 nm (ex/em) for Hoechst 33342 and DCFH-DA, respectively, as explained above.

### 2.6. Integrity of lysosome and mitochondria

Lysosomal and mitochondrial integrities were determined via Neutral Red (NR) and Janus Green B (JGB) staining in the presence of Hoechst 33342 dye [22–24]. The cells were seeded and incubated with DEHP, or solvent, as described above, and fixed with 4% paraformaldehyde. After washing, the fixed cells were further incubated with NR (1 μM; Sigma-Aldrich Chemical Co.) and JGB (5 μM; Sigma-Aldrich Chemical Co.) for 30 min at 37 °C. After a single washing with PBS, the cells were further treated with Hoechst 33342 (1 μg/mL) for 5 min at room temperature. Finally, images were obtained at 361/497 nm (ex/em; Hoechst), 550/655 nm (ex/em; NR), and 490/516 nm (ex/em; JGB), as explained above.

### 2.7. Ferritin and CD11b immunoreactivity

Immunocytochemistry was performed to assess the levels of ferritin heavy chain 1 (FTH1) and CD11b [25,26]. After seeding, treatment with DEHP or solvent, and fixing, the cells were treated with 0.25% Triton X-100 in PBS (PBST) for 5 min. After washing, the cells were blocked with PBST containing 3% bovine serum albumin (BSA) for 45 min and incubated with rabbit antimouse ferritin (1:200; Thermo Fisher Scientific Inc.) or anti-CD11b antibody (1:100; Thermo Fisher Scientific Inc.) at 4 °C overnight. After washing, the cells were treated with goat antirabbit IgG-CFL 488 secondary antibody (1:1000; Santa Cruz Biotechnology Inc., Dallas, TX USA) for 45 min at room temperature. After washing and treatment with Hoechst 33342 (1 μg/mL) for 5 min at room temperature, images were obtained at 361/497 nm (ex/em; Hoechst) and 361/488 nm (ex/em; CFL-488), as explained above.

### 2.8. IL-1β and IL-18 levels

Enzyme-linked immunosorbent assay (ELISA) was used to determine the interleukin 1β (IL-1β) and IL-18 levels using commercial mouse ELISA kits (Thermo Fisher Scientific Inc.). After seeding the cells into 6-well plates, treated with DEHP or solvent, for the specified incubation periods, the mediums on the cells were collected in 1.5-mL tubes. Next, the samples were centrifuged at 12,000 g for 15 min at 4 °C, and supernatants were obtained. ELISA analyses were performed according to the manufacturer’s instructions. In brief, 100 μL of the supernatants were dispended in antibody-embedded 96-well plates, and after all the steps were performed, absorbance was read at 450 nm using a microplate reader. All the measurements were made according to the kits’ standard curve graphs (per se) and normalized with their protein concentrations.

### 2.9. Expression levels of both iron metabolism and ferroptosis-related proteins

Quantitative reverse-transcriptase polymerase chain reaction (qRT-PCR) was performed to determine the relative expression levels of iron metabolism genes, including *Dmt1* (divalent metal transporter 1), ferroportin *(Fpn*), ferritin heavy chain 1(*Fth1*) and ferritin light chain 1 (*Ftl1)* and as well as ferroptosis-related proteins such as glutathione peroxidase 4 (*Gpx4)* and Acyl-CoA synthetase long-chain family member 4 *(Ascl4)*.

After seeding the cells and the treatments, the total RNAs were isolated using a Trizol reagent (Thermo Fisher Scientific Inc.). Briefly, the cells were treated with 1 mL of Trizol, mixed well, and incubated at room temperature for 5 min. Next, 200 μL of chloroform was added to the samples, and the mixture was incubated on ice for 10 min. The samples were centrifuged at 12,000 g at 4 °C for 15 min to obtain pellets. The pellets were washed with 75% cold ethanol and 0.5 mL of isopropanol. The final pellets were resuspended with 100 μL of RNase-free water. The concentration and purity of the total RNAs were determined using a Nanodrop (ND-1000; Thermo Fisher Scientific Inc.) and 1% agarose gel.

Complementary DNAs (cDNAs) were synthesized using a Hyperscript First strand synthesis kit (GeneAll Biotechnology Co., Ltd., Songpa-gu, Seoul, South Korea). The reactions were prepared using 1 μg of total RNA, oligo dT, dNTP set, buffer, RNase inhibitor, dithiothreitol (DTT), and reverse transcriptase according to the kit’s instructions. The mixture was incubated at 55 °C for 1 h. Finally, the reverse transcriptase was inhibited by EDTA and incubated at 85 °C for 5 min.

After cDNA synthesis, qRT-PCR was performed using SYBR Green solution (GeneAll Biotechnology Co., Ltd.). The primer sets are listed in the Table, where peptidylprolyl isomerase A (*Ppia)* was used as a reference gene. qRT-PCR was performed using 5 μL of SYBR Green mix, 0.5 μL of forward and reverse primers (10 μM), and 4 μL of diluted (1:20) cDNAs. The thermal profile was set to 95 °C for 5 min as the initial denaturation; cyclic (n = 40) denaturation, annealing, and extension, at 95 °C for 30 s, 60 °C for 30 s, and 72 °C for 30 s, respectively; and 72 °C for 10 min as the final extension. The qRT-PCR was stopped by the addition of a melting curve step at a cycle of 55–99 °C. Each sample was in the presence of a technical replica (n = 3). All the cycle threshold (Ct) values were evaluated using the 2^(−delta delta Ct) method [27].

### 2.10. Inhibition of ferroptosis

To further understand the mechanistic involvement of ferroptosis, this death pathway was blocked by ferroptosis inhibitor ferrostatin-1 (Sigma-Aldrich Chemical Co.). Cells were seeded into the wells of a 96-well plate and incubated with a half-maximal inhibitory concentration (IC_50_) of DEHP in the presence or absence of different concentrations (0.5–50 μM) of ferrostatin-1. Subsequently, an MTT assay was performed, as explained above.

### 2.11. Statistical analyses

All the experiments were repeated as proper technical and biological replicas (n = 9). Statistical analyses were performed using GraphPad Prism for Windows 8.0 (Boston, MA, USA) program. All the data were expressed as the mean ± standard deviation (SD). Data were analyzed using one-way analysis of variance (ANOVA) or the Kruskal–Wallis method with appropriate post hoc tests. The results were considered significant at p < 0.05.

## 3. Results

### 3.1. Effect of DEHP toxicity on apoptotic/necrotic death in BV-2 cells

**Figure 1:**
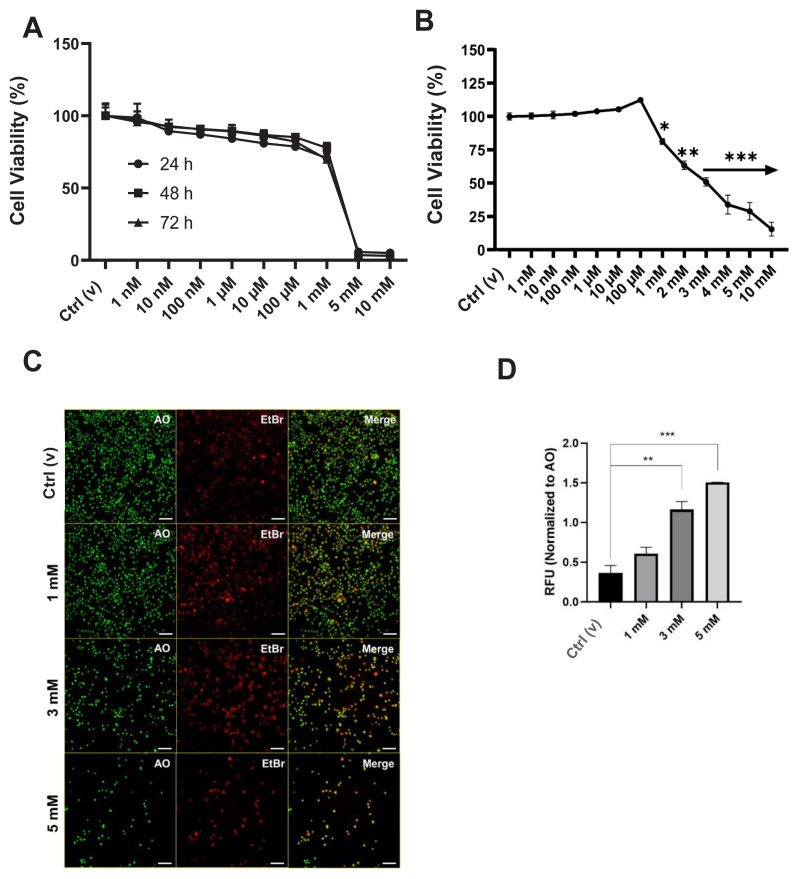
Effect of DEHP on cellular viability. A. Cell viability by DHEP treatment on BV-2 cells with increasing concentrations (1 nM–10 mM) for 24, 48, or 72 h. B. Expanded cell viability observed by applying DEHP for 24 h. The arrow shows that the remaining concentrations are of the same statistical significance level. C. Evaluation of the apoptotic/necrotic cells by the application of DEHP. D. Quantification of the relative fluorescence with reference to C. RFU: Relative integrated fluorescence unit; Green, AO: Healthy; Red, EtBr: apoptotic/necrotic. Scale: 50 μm. *p < 0.05, **p < 0.01, ***p < 0.001 (ANOVA, post hoc Dunnet’s test).

The results of the MTT assay were as follows: DEHP at concentrations exceeding 1 mM was highly toxic, as illustrated in Figure 1A, regardless of the incubation period. The MTT experiment yielded the following results: DEHP at concentrations greater than 1 mM exhibited significant toxicity. This observation, excluding the incubation duration, is depicted in Figure 1A. The IC_50_ was 3 mM (Figure 1B).

As an underlying molecular mechanism, the apoptotic/necrotic cell death of BV-2 cells was illustrated by fluorescence imaging using AO and EtBr staining, which revealed [17] an elevated EtBr signal with 3 and 5 mM of DEHP (Figures 1C and 1D). EtBr fluorescence at a concentration of 1 mM increased significantly compared to the control (Figure 1D).

### 3.2. Effect of DEHP on inflammation in BV-2 cells

**Figure 2:**
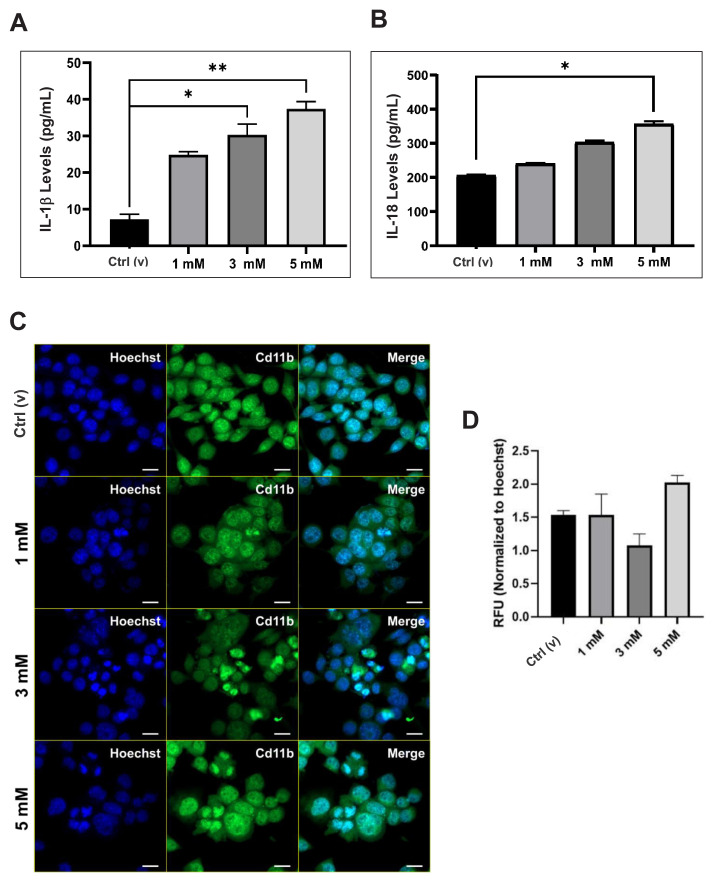
Effect of DEHP on neuroinflammation. A and B. Changes in IL-1β and IL-18 levels by the DEHP treatment. C. CD11b expression by DEHP treatment. D. Quantification of the relative fluorescence with reference to C. Blue, Hoechst 3342: Nucleus; Green, CFl-488: CD11b. Scale: 20 μm. *p < 0.05 and **p < 0.01 (Kruskal–Wallis post hoc Dunn’s test).

The ELISA test results showed that 3 and 5 mM of DEHP increased the IL-1β levels significantly. However, only 5 mM of DEHP increased the IL-18 levels significantly (Figures 2A and 2B). The CD11b expression increased with 5 mM of DEHP, even though it was not statistically significant (Figures 2C and 2D), indicating CD11b’s regulatory effect and proinflammatory nature [28]. These results showed the dose-dependent proinflammatory effect of DEHP.

### 3.3. Effect of DEHP on intracellular iron levels and metabolism

**Figure 3:**
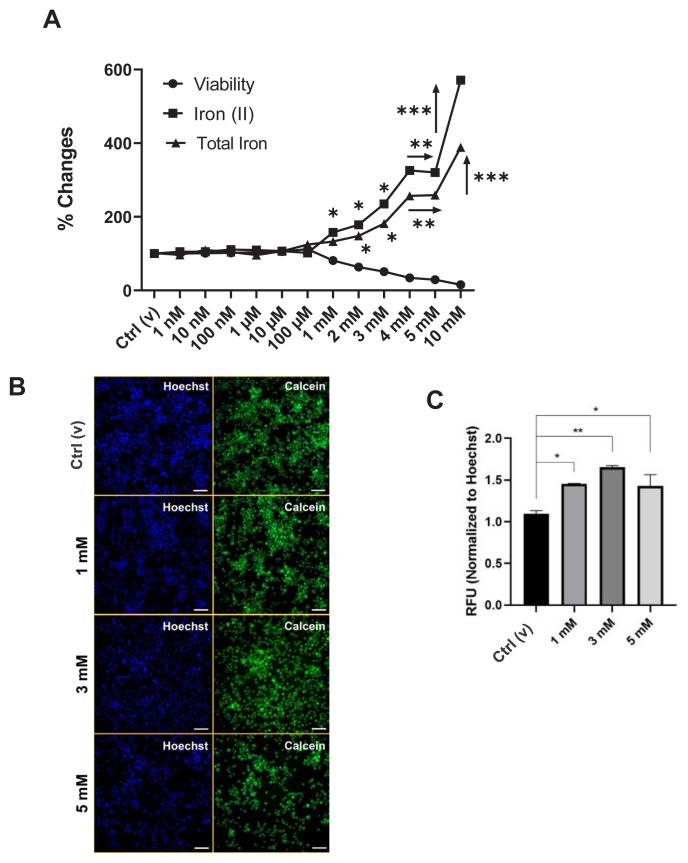
Effect of DEHP on the cellular iron levels. A Changes in intracellular iron levels caused by the application of DEHP to BV-2 cells with increasing concentration (1 nM–10 mM) for 24 h. B. Confocal images of the labile iron pool (LIP) after applying DEHP. C. Quantification of the relative fluorescence with reference to B. RFU: Relative integrated fluorescence intensity. Blue, Hoechst 3342: Nucleus; Green, Calcein-AM: LIP. Scale: 50 μm. Arrows show that the related concentrations are of the same significance level. *p < 0.05, **p < 0.01, and ***p < 0.001 (Kruskal–Wallis post hoc Dunn’s test).

**Figure 4:**
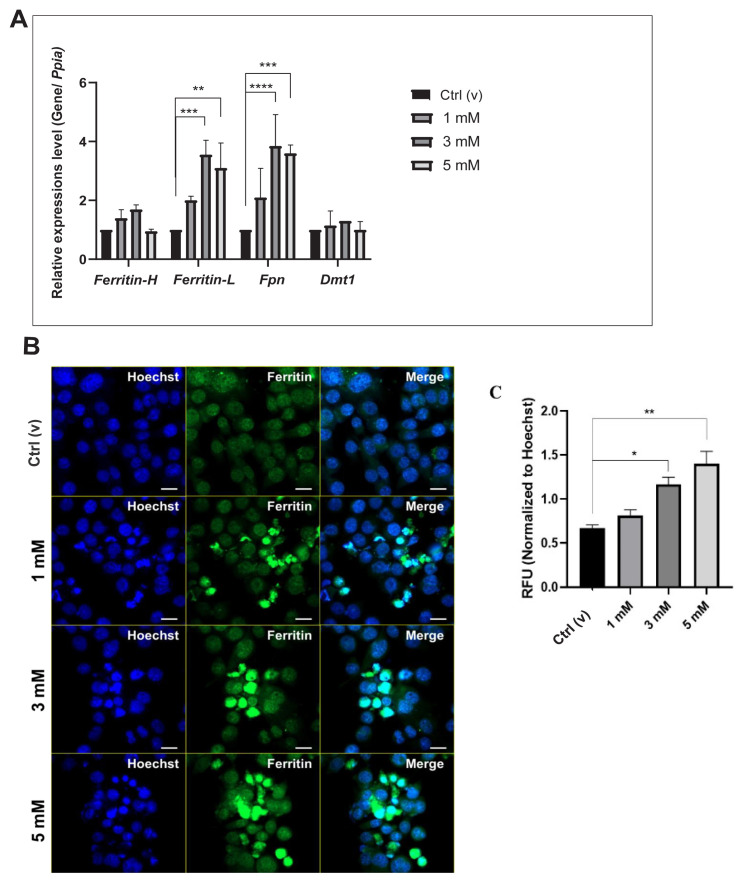
Effect of DEHP on iron-metabolism-related proteins. A. Expression levels of iron metabolism-related proteins by DEHP administration at different concentrations in BV-2 microglia cells. B. Change in ferritin immunoreactivity by applying DEHP. C. Quantification of relative fluorescence in the B. Blue, Hoechst 3342: Core; Green, CFl-488: Ferritin. Scale: 20 μm. **p < 0.01, ***p < 0.001, and ****p < 0.0001 (ANOVA).

The cellular iron levels were checked following treatment with DEHP for molecular characterization. Both the total and Fe^2+^ iron levels significantly increased due to the toxic concentrations of DEHP (Figure 3A). Moreover, the LIP was also elevated according to the Calcein-AM staining (Figures 3B and 3C). The expression of iron storage (*Fth1 and Ftl1)*, exporter *(Fpn*), and importer (*Dmt1*) genes determined by the qRT-PCR and ferritin levels determined by immunohistochemistry increased (Figures 4A–4C), while the importer protein was stable with 3 and 5 mM of DEHP (Figure 4A).

### 3.4. Effect of DEHP-induced elevated intracellular ROS levels on organelles

**Figure 5:**
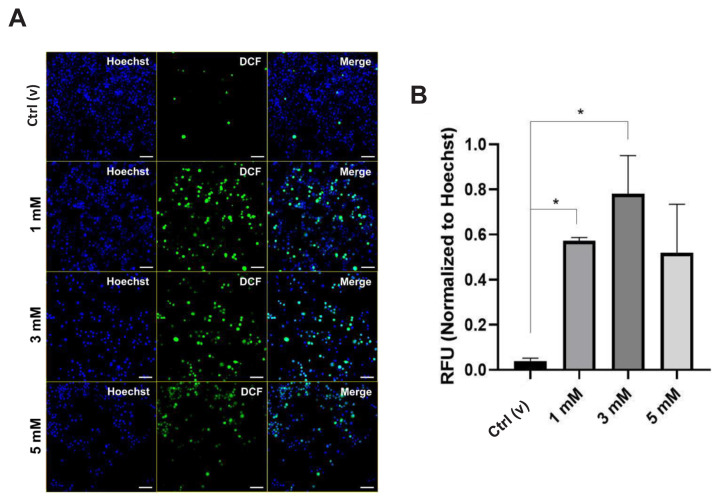
A. Changes in the intracellular reactive oxygen levels by applying DEHP to BV-2 cells. B. Quantification of the relative fluorescence with reference to A. Green, DCF: Dichlorofluorescein; Blue, Hoechst (Nucleus). Scale: 50 μm.

The results showed increased ROS levels with 1 and 3 mM of DEHP (Figures 5A and 5B). Underlying DEHP caused putative ROS generation via intracellular iron accumulation. Although the ROS levels relatively increased with 5 mM of DEHP, it was not statistically significant (Figures 5A and 5B). However, elevated iron, as proven by detecting the cellular iron levels and changing iron metabolism-related protein levels, can produce ROS to initiate oxidative stress [29].

**Figure 6:**
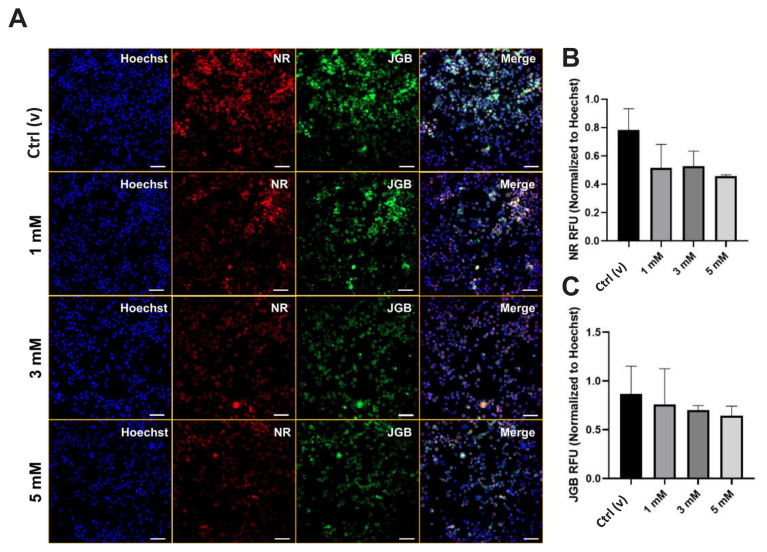
Confocal images of lysosomal and mitochondrial integrity as a result DEHP treatment. B. Quantification of the relative NR fluorescence with reference to A. C. Quantification of the relative JGB fluorescence with reference to A. Red, NR: Lysosome; Green, JGB: Mitochondria; Blue, Hoechst 3342: Nucleus. Scale: 50 μm.

Following cellular characterization, mitochondrial and lysosomal integrity was examined via fluorescence staining. NR is a dye that can assess lysosomal integrity, while JGB can be used to determine mitochondrial integrity. Decreases in the fluorescence signals for these dyes point to defects in the lysosomal or mitochondrial integrity [30]. The results herein showed that there were no significant alterations in the fluorescence signals (Figure 6A). Nevertheless, the DEHP treatment decreased the fluorescence signals coming from both NR (Figure 6B) and JGB (Figure 6C) at high concentrations, manifesting the possible defects in both lysosomal and mitochondrial integrity, which were crucial for any cell death mechanisms [31] and vice versa.

### 3.5. Effect of DEHP on ferroptosis-related gene expression and ferroptosis

**Figure 7:**
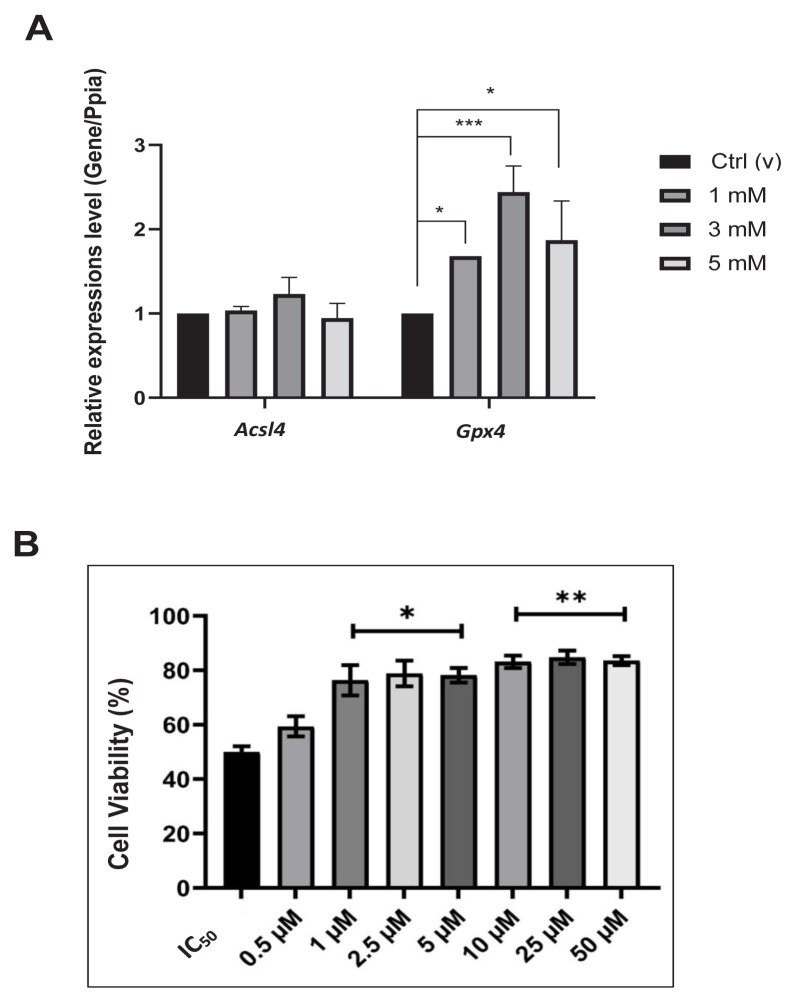
Effect of DEHP on ferroptosis. A. Expression levels of ferroptosis-related proteins by DEHP administration at different concentrations in BV-2 microglia cells. B. Effects of ferrostatin-1 treatment at increasing concentrations (0.5–50 μM) on cell viability. *p < 0.05, **p < 0.01, and ***p < 0.001 (ANOVA and Kruskal–Wallis posthoc Dunn’s test).

The results illustrated that the expression of Gpx4 increased, while that of Ascl4 was not altered by the DEHP (Figure 7A). Ferroptosis was further analyzed using a ferroptosis inhibitor, ferrostatin-1, in the presence of DEHP. The results revealed that ferrostatin-1 blocked DEHP-dependent cell death (Figure 7B), confirming DEHP-dependent ferroptosis in BV-2 cells.

## 4. Discussion

The present study aimed to interpret the possible effects of phthalates on neonatal microglial cells. After DEHP exposure, it was aimed to reveal the molecular mechanisms underlying the neurotoxicological pathways involved in neuroinflammation by focusing on oxidative damage, inflammatory markers, and diverse cell death types. The results showed that DEHP at high concentrations caused elevated levels of ROS and inflammation, resulting in cellular toxicity in the BV-2 cell line. Moreover, high concentrations of DEHP promoted the loss of mitochondrial and lysosomal integrity, underlying the induction of apoptotic or necrotic cell death mechanisms. Finally, the ferroptotic cell death mechanism was also initiated by DEHP exposure through dysregulated iron metabolism.

Phthalates have been identified as potential endocrine disruptors that could result in various pathologies, including hypospadias, cryptorchidism, decreased anogenital distance, growth and pubertal problems during childhood, obesity, insulin resistance, type 2 diabetes, endometriosis, breast cancer, respiratory and allergic diseases, and neurodevelopmental and neurobehavioral developmental disorders [32–34]. The European Union enforced a prohibition on the utilization of proscribed DEHP, di-iso-butyl phthalate (DiBP), and butyl-benzyl phthalate (BBzP) in toys, childcare articles, and cosmetics in a 2009 (MDR 2017/745) regulation, stating that medical devices containing carcinogenic, mutagenic, or toxic for reproduction 1A/1B and endocrine disruptive compounds substances above a concentration of 0.1 weight percent must be justified and labelled accordingly [35]. Nonetheless, multiple studies carried out in NICUs have reported that exposure of infants to DEHP could exceed this ratio [36].

The perinatal period could be riskier for exposure to DEHP, resulting in drawbacks in development, including neurodevelopmental stages [6,37–39]. In large population studies of DEHP-induced neurotoxicity, the environmental phthalate exposures of pregnant women were evaluated based on their urine levels. The neurodevelopmental scores of children showed that higher exposure to phthalate metabolites caused lower neurodevelopmental scores and behavioral problems, including autism spectrum disorders [37,40,41]. However, the precise cellular and molecular pathways underlying the in vivo effects of phthalates remain incompletely elucidated.

In the present study, DEHP was chosen to evaluate the possible effects of phthalates on the BV-2 cell line. Among phthalates, DEHP is critically dangerous for neonates, as it is widely used in the medical equipment in NICUs [3,6,35]. Previous studies have underlined that DEHP causes elevated ROS levels, the induction of apoptosis, and altered epigenetic mechanisms in diverse neuronal cell lines [15,42]. In the present study, the viability assay showed that DEHP was remarkably toxic, regardless of the incubation period when the concentrations were above 1 mM (Figures 1A and 1B). Studies with human cell lines have underlined that although lower concentrations of DEHP were not toxic in a time-dependent manner, higher concentrations (0.2–0.5 mM) were [43], confirming these results.

To determine the possible cell death mechanisms, apoptosis and necrosis were examined herein. The findings highlighted the induction of apoptosis and necrosis in high-dose DEHP-treated cell groups when compared to the control (Figures 1C and 1D). The induction of apoptosis by DEHP in neuronal cell lines has been well documented using apoptotic markers such as cleaved caspases and Bax/Bcl2 ratios [15]. EtBr staining aids in identifying damaged cell membranes as a sign of apoptosis [17]. However, we propose that both apoptosis and necrosis are involved because membrane disruption is common in both types of cell death when DEHP levels are high. Increased immunologic activity at higher DEHP concentrations (Figures 2A–2D), as previously reported, may also be linked to apoptosis [44].

Iron levels are critical for brain development in the prenatal and early neonatal periods. Maintaining iron homeostasis is crucial for ensuring proper cellular function. This regulation involves a complex interplay of various transport proteins responsible for different aspects of iron metabolism. DEHP-mediated ROS generation has been reported to be neurotoxic [11], and ferroptosis, which alters iron metabolism and triggers the production of ROS and reactive lipid species [45,46], could also be dominant for microglia. According to the results of the current study, the DEHP treatment elevated both the iron uptake and storage, which were shown by the cellular free and total iron (Figure 3A) and LIP levels (Figures 3B and 3C). Iron is internalized by the cells in ferric (Fe^3+^) form and induced and stored in Fe^2+^ form [10]. Cellular iron regulation is supported by iron import (transferrin-transferrin receptor 1 (Tf-TfR1)), intracellular transport (Dmt1), storage (ferritin light and heavy chain; Ftl and Fth), and export (ferroportin; Fpn) proteins [46]. The administration of 1 and 3 mM of DEHP significantly increased the Ftl and Fpn levels, while no significant alteration was observed in Fth and Dmt1 levels (Figure 4A). The increase in the expression of the exporter protein in the present study would be a compensation strategy, as shown in our previous studies [10,26,47].

Moreover, ferritin immunoreactivity increased after DEHP administration, accumulating in the cell when cell integrity was compromised (Figures 4B and 4C). As previously shown, iron uptake was increased by the DEHP treatment via modulation of the expression of the iron transport proteins. Although one study showed the expression of iron storage proteins was reduced and those of uptake proteins were upregulated in the liver cells [48], another study illustrated that the expression of storage proteins increased in the spleen cells [49] by the DEHP exposure. Hence, the differences could be tissue-specific.

When there is an excessive build-up of iron within the cellular environment, it can lead to the generation of detrimental free radicals, primarily mediated by iron. These free radicals can cause oxidative damage and disrupt normal cellular processes. Thus, the intracellular ROS levels were quantified herein and it was proven that the ROS levels were high with all the treatments, although it was not significant with 5 mM of DEHP (Figures 5A and 5B).

Elevated ROS levels following DEHP treatment has frequently been reported [14], validating our results. DEHP-dependent ROS-mediated mitochondrial alterations and, thus, apoptosis has also been underlined [50]. Therefore, the integrities of cell death-related organelles, mitochondria, and lysosomes were evaluated. The results illustrated that the integrities of those organelles were slightly decreased, even though it was not statistically significant (Figures 6A–6C), confirming the partial involvement of apoptosis. ROS generation by DEHP treatment and its possible effects gained great attention in different cell models [3,13].

The effect of excessive ROS generation has frequently been studied in cell death mechanisms, including ferroptosis [46]. Ferroptosis by DEHP-mediated ROS generation has also been studied in neonates; however, these studies mainly centered on sexual development [48,51]. Hence, the current study focused on the effects of DEHP treatment on the neonatal microglia, particularly concentrating on the ferroptotic cell death mechanism. This study evaluated the effects of varying concentrations of DEHP on the expression of two key regulators of ferroptosis, Acls4 and Gpx4. The increased levels of DEHP did not result in a corresponding increase in the expression of Acls4; however, there was a notable increase in the expression of Gpx4 (Figure 6A).

Similarly, in one study, even with increasing dosages of DEHP, the expression of Acsl4 in BV-2 microglia remained unchanged [52]. These results indicate that distinct enzyme pathways are likely to be involved in regulating ferroptosis in microglia. To further evaluate the ferroptotic pathways, ferroptosis was blocked in the present study using ferrostatin-1, which mainly inhibits autooxidation and nonenzymatic destruction of the membrane via polyunsaturated-fatty-acid (PUFA) containing phospholipids driven by Fenton chemistry during ferroptosis [53]. In response to metabolic activities that trigger ferroptosis, cells have evolved diverse defense mechanisms to inhibit the generation of cytotoxic lipid peroxides and promote cell survival. The Gpx4 signaling axis is the principal cellular defense mechanism against ferroptosis [54]. The current research results indicated that adding a ferroptosis inhibitor led to a 25% increase in cell viability (Figure 7B).

Furthermore, as the inhibitor dosage was increased, the cell viability showed a further increase of 30%–35%. Despite administering a ferroptosis inhibitor, the lack of a significant increase in cellular survival rates implies that alternative forms of programmed cell death, such as pyroptosis, may occur when assessed concurrently with increased inflammatory markers. The observed correlation between pyroptosis and the elevated levels of IL-1β and IL-18, along with increased free iron within the cell, suggests that phthalates may induce a multistep mechanism of cell death in the developing brain.

**Figure 8:**
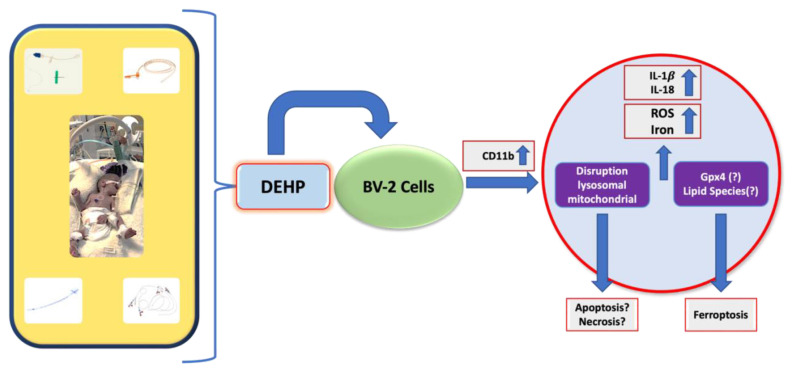
Proposed effects of DEHP on neonatal microglia from a clinical to molecular perspective.

The results herein showed that exposure to DEHP increased ROS and iron accumulation, disrupted the organelle structures, and caused neuroinflammation, apoptosis, and ferroptosis (Figure 8). Given that distinct subtypes of cell death retain their specific characteristics, and the underlying processes vary, crosstalk or links may occur between the various cell death pathways. Even while the lysosomal alterations in the present study mostly resulted in ferroptosis, the increase in IL-1β and IL-18 implied that another inflammation-related route may also be active or there may be a connection between cell death pathways. As a component used in medical devices, particularly in NICUs, DEHP exposure to neonates should be strictly limited. Moreover, further molecular and in vivo studies are needed, especially to fully explore the involvement of the ferroptotic cell death mechanisms by exposure to DEHP.

## Figures and Tables

**Table:** List of primers used in the qRT-PCR.

Gene	Forward primer	Reverse primer
*Dmt1*	5′TGAATCGGCCAATAAGCAGGCA3′	5′ATCAGCAAAGACGGACACGACAA3′
*Fpn*	5′AGAGCTGACCTGGCACCTTA3′	5′GGCCCAAGTCAGTGAAGGTA3′
*Fth1*	5′TAAAGAACTGGGTGACCACGTGAC3′	5′AAGTCAGCTTAGCTCTCATCAGCG3′
*Ftl1*	5′TGGCCATGGAGAAGAACCTGAATC3′	5′GCTTTCCAGGAAGTCACAGAGAT3′
*Gpx4*	5′TAAGAACGGCTGCGTGGTGAAG3′	5′AGAGATAGCACGGCAGGTCCTT3′
*Ascl4*	5′CCTTTGGCTCATGTGCTGGAACT3′	5′CAGCGGCCATAAGTGTGGGTTT3′
*Ppia*	5′CCCACCGTGTTCTTCGACAT3′	5′CCAGTGCTCAGAGCACGAAA3′
